# From Macro to Mesoporous ZnO Inverse Opals: Synthesis, Characterization and Tracer Diffusion Properties

**DOI:** 10.3390/nano11010196

**Published:** 2021-01-14

**Authors:** Shravan R. Kousik, Diane Sipp, Karina Abitaev, Yawen Li, Thomas Sottmann, Kaloian Koynov, Petia Atanasova

**Affiliations:** 1 Institute for Materials Science, University of Stuttgart, 70569 Stuttgart, Germany; shravan.ravishankar-kousik@imw.uni-stuttgart.de (S.R.K.); diane.sipp@gmail.com (D.S.); yawen.ywl@outlook.com (Y.L.); 2 Institute of Physical Chemistry, University of Stuttgart, 70569 Stuttgart, Germany; karina.abitaev@ipc.uni-stuttgart.de (K.A.); thomas.sottmann@ipc.uni-stuttgart.de (T.S.); 3 Max Planck Institute for Polymer Research, 55128 Mainz, Germany

**Keywords:** inverse opals, mesoporous materials, ZnO, diffusion in pores, fluorescence correlation spectroscopy

## Abstract

Oxide inverse opals (IOs) with their high surface area and open porosity are promising candidates for catalyst support applications. Supports with confined mesoporous domains are of added value to heterogeneous catalysis. However, the fabrication of IOs with mesoporous or sub-macroporous voids (<100 nm) continues to be a challenge, and the diffusion of tracers in quasi-mesoporous IOs is yet to be adequately studied. In order to address these two problems, we synthesized ZnO IOs films with tunable pore sizes using chemical bath deposition and template-based approach. By decreasing the size of polystyrene (PS) template particles towards the mesoporous range, ZnO IOs with 50 nm-sized pores and open porosity were synthesized. The effect of the template-removal method on the pore geometry (spherical vs. gyroidal) was studied. The infiltration depth in the template was determined, and the factors influencing infiltration were assessed. The crystallinity and photonic stop-band of the IOs were studied using X-Ray diffraction and UV-Vis, respectively. The infiltration of tracer molecules (Alexa Fluor 488) in multilayered quasi-mesoporous ZnO IOs was confirmed via confocal laser scanning microscopy, while fluorescence correlation spectroscopy analysis revealed two distinct diffusion times in IOs assigned to diffusion through the pores (fast) and adsorption on the pore walls (slow).

## 1. Introduction

Inverse opals (IOs) are a class of 3-dimensionally ordered macroporous (3DOM) materials [1,2] that have been extensively studied for their application as 3-D photonic crystals [3,4,5], biosensors [6,7,8] and photocatalysts [9,10,11]. The fabrication of oxide IOs generally involves the incorporation of a metal oxide into an opaline polymer colloidal crystal template through physical or chemical techniques such as electrodeposition [12], atomic layer deposition [13], ultrasonic spray pyrolysis [14], sol-gel [15,16] and hydrothermal synthesis [17]. There are mainly two approaches that can be utilized to fabricate oxide IOs—co-assembly and mineralization. The former involves concurrent assembly of the polymeric colloidal crystal template and the desired oxide nanoparticles. In the latter, the polymeric template particles are first assembled into an opaline matrix and then a metal oxide precursor is infiltrated into its voids and allowed to mineralize. In both cases, the template is removed after assembly by calcination or extraction with an organic solvent. Amongst the many metal oxides that have been considered for photonic crystal applications, ZnO is of particular significance. In addition to its high thermal and mechanical stability [18], its wide band gap (3.37 eV) [19], large exciton binding energy [20] and strong room-temperature luminescence [21] render it a promising candidate for applications such as short-wavelength LEDs [22] and UV-lasing [23,24,25].

In recent years, the use of metal oxides as solid supports for molecular heterogeneous catalysis has seen an upward surge [26,27,28,29]. In such catalytic processes, active metal nanoparticles [30,31,32], coordination complexes [33,34,35] and enzymes [36,37] are immobilized on the support material through anchor groups or functionalized linker molecules, self-assembled on the pore walls. Although metal oxides can be fabricated with varying morphologies, IOs possess distinct advantages vis-à-vis catalysis. The hierarchical porosity, offered by IOs, ensures enhanced surface areas for the attachment of catalyst molecules. Additionally, the presence of open pores facilitates the diffusion of reactant molecules through the porous support [38]. Furthermore, the occurrence of confined domains in the mesopore range (2–50 nm) can impart enhanced stereo and regioselectivity during the catalytic reaction. The ability to tune the pore sizes of IOs makes them a viable candidate for catalytic support applications [39]. It is to be noted that the efficiency of heterogeneous catalysis is largely dependent on the diffusion and distribution of reactant molecules within the solid support [40]. Quantifying the diffusion behavior of analytes in solid supports is therefore an incipient area of research. Although techniques like XPS and contact angle measurements have been used to determine the existence of self-assembled monolayers (SAMs) on IO pore walls, they cannot be applied to quantitively study the diffusion and distribution of probe molecules within porous scaffolds [41,42]. Mapping the immobilization of SAM and catalyst molecules within metal oxide IOs would allow for the synthesis of targeted catalyst-support combinations with improved activity and selectivity. A powerful technique that can be used to study molecular transport in porous systems is confocal laser scanning microscopy (CLSM). This method was successfully used to map the distribution of biological analytes such as cells and enzymes on inverse opals [43,44]. Besides monitoring the localization of probe molecules on porous catalytic supports, there is also an overwhelming need to study their transport properties. The combination of CLSM with fluorescence correlation spectroscopy (FCS) [45] would enable us to visualize the distribution of catalysts in porous materials and at the same time, obtain real-time information on vital parameters such as diffusion, permeation efficiencies and infiltration depths [46,47,48,49,50].

From a synthetic standpoint, wet-chemical templating of IOs has gained prominence due to its versatility and cost-effectiveness. However, the fabrication of inverse opals with mesoporous or sub-macroporous voids (<100 nm) continues to be a challenge. In addition, the rapid crystallization of ZnO at room temperatures [51] and the calcination-induced shrinkage of the opaline matrix [1] greatly inhibit the ability to synthesize high-quality ZnO IO films. In order to preserve the monodispersity of the template and the ensuing IO, most sol-gel based templating methods employ colloidal crystal templates whose particle diameters are in the range of hundreds of nanometers (Table 1). Efforts to reduce the void sizes of inverse opals through multiple mineralization cycles are also at a dormant stage. Although methods like colloidal self-assembly of precursors, gravimetric sedimentation and chemical vapor deposition have been used to synthesize SiO_2_ IOs in bulk [52,53], the same cannot be applied for ZnO. The techniques that have been used thus far for the synthesis of ZnO IOs in large quantities involve complex procedures in which acetate precursors are converted into oxalate salts in-situ followed by pyrolytic decarboxylation to yield the resultant oxide IO. This approach again is restricted to colloidal crystal templates with particle diameters in the range of 620–760 nm [54]. Although a large corpus of information is available on the sol-gel synthesis of ZnO IOs with macroporous voids, very few attempts have been made to extend this approach to the mesoporous domain. It is therefore incumbent upon us to develop a technique that can be used to produce ZnO IO films with tunable pore sizes.

In this work, ZnO IOs films with tunable pore sizes in the macro-mesoporous range have been fabricated via a template-based method. Polystyrene (PS) particles with varying diameters were assembled as opaline template films, and then, infiltrated with a ZnO precursor solution, the polymer template was mineralized through a relatively simplistic chemical bath deposition (CBD) method. Using PS template particles with decreasing diameters (175–48 nm), the pore size was reduced towards the mesoporous range. Further pore size reduction was attempted by subjecting the synthesized ZnO IOs to additional deposition cycles of ZnO on the pore walls. The infiltration depth of the ZnO precursor in template films, fabricated with diverse PS particles, has been systematically studied. The effects of the template removal process on the pore geometry and integrity of the ZnO IO films have been summarily demonstrated. Furthermore, the infiltration of tracer molecules within ZnO IO films was tracked via CLSM, while their diffusion properties in the porous oxide structure have been quantified using FCS.

## 2. Materials and Methods

### 2.1. Synthesis of PS Particles

PS nanoparticles were synthesized by emulsion polymerization of styrene under inert gas, using potassium peroxodisulfate (KPS) as initiator and the emulsifier sodium dodecyl sulfate (SDS), according to literature [55]. Briefly, a mixture of styrene (21 mL) (Sigma-Aldrich, Taufkirchen, Germany) and 2-ethyl hexyl thioglycolate (0.5 mol%) (Sigma-Aldrich) was added to an 80 °C pre-heated solution of degassed double-distilled water (180 mL) and a certain amount of SDS (Sigma-Aldrich) placed in a three-necked round bottom flask. The emulsion was stirred at 750 rpm for 10 min, prior to the addition of the KPS (Sigma-Aldrich) solution (0.05 mM in 10 mL double-distilled, degassed water). The temperature and stirring speed were kept constant for additional 4.5 h, resulting in a turbid dispersion with ~10 wt% solid content. In this way, PS particles with different diameters (~175 nm, ~70 nm, ~60 nm and ~48 nm) were synthesized adjusting the surfactant concentration to 5.0 mM, 6.5 mM, 8.0 mM and 13.1 mM, respectively, while all other parameters were kept constant. The syntheses of PS particles, performed at constant surfactant concentration, was reproducible yielding particles with very similar diameters (*d_part_*/<*d_part_*> ≈ ±0.07) and particle size distributions (*σ_part_*/<*σ_part_*> ≈ ±0.02). For the ZnO IO assemblies produced in this work, PS colloidal dispersions with the corresponding particle sizes (175, 70, 60 and 48 nm) were prepared once, and the same dispersions were used for all experiments.

### 2.2. Substrate Cleaning

For our experiments, polished p-type (100) silicon substrates (Siltronic AG, Munich, Germany) and glass slides (Paul Marienfeld GmbH, Lauda-Königshofen, Germany) were used. The substrates and glass slides were cleaned and hydrophilized as follows: (1) 10 min sonication in ultrapure water, (2) 10 min sonication in ethanol/acetone (1:1 *v*/*v*), (3) 10 min O_2_ plasma treatment (at 30 W) to burn off organic impurities and hydrophilize the substrate and (4) 10 min sonication in ultrapure water. After each sonication step, the substrates were washed 10 times with the corresponding solvent and dried under N_2_.

### 2.3. Preparation of Hydrophobic Glass Slides for Convective Assembly

15 µL of perfluorodecyltriethoxysilane (Sigma-Aldrich) was added to the bottom of a desiccator. O_2_ plasma-treated glass slides (2 × 2 cm^2^) were affixed to the lid of the desiccator using a Kapton tape. The desiccator was then evacuated using a vacuum line until a pressure of 0.05 mbar was reached, and then, the desiccator was closed. The evaporation of the silane upon evacuation was confirmed from the appearance of bubbles at the bottom of the desiccator. The hydrophobic silane was then allowed to self-assemble on the glass slides for 24 h. After assembly, the hydrophobicity of the glass slides was analyzed using contact angle measurements.

### 2.4. Assembly of PS Template Films

Colloidal dispersions of PS (10 wt%) with different diameters (~175, 70, 60 and 48 nm) were assembled into thin films on plasma cleaned Si wafers (1.5 × 1.5 cm^2^) via convective assembly. 10 µL of the desired PS dispersion was drop-cast on the triple contact line air-silicon substrate-hydrophobic glass slide positioned on an improvised convective assembly setup as described in [56]. For the assembly of the PS particles on the substrate, withdrawal speeds ranging from 0.1–0.6 mm/min were used. The assembly was carried out at ambient temperature (21–23 °C) and at a relative environmental humidity of 35–55%**.** The template films were labelled as follows: PS175-T (template film prepared with ~175 nm PS particles), PS70-T (with ~70 nm PS), PS60-T (with ~60 nm PS), and PS48-T (with ~48 nm PS).

### 2.5. ZnO Mineralization

The ZnO deposition was carried out in accordance with a previously established procedure [57]. Briefly, stock methanolic solutions of Zn(OOCCH_3_)_2_•2 H_2_O (34 mM) (Sigma-Aldrich), tetraethylammonium hydroxide (TEAOH) (75 mM) (Aldrich Chemistry) and polyvinylpyrrolidone (PVP) (21.7 mM) (Sigma-Aldrich, M_w_ = 10,000, Lot # BCBJ4889V) were prepared. The actual precursor solutions were prepared by mixing one volume unit of zinc acetate with one volume unit of PVP stock solutions. Then, one volume unit of TEAOH stock solution was added dropwise via a peristaltic pump at a flow rate of 1.04 mL min^−1^ under continuous stirring. The substrates to be mineralized were placed in vessels with 1 mL of the precursor solution and heated in an oil bath at 60 °C for 1.5 h (1 deposition cycle). For subsequent mineralization cycles, the substrates were removed from the precursor solution, washed thoroughly with methanol, dried under an inert gas stream and reintroduced into a fresh aliquot of the precursor solution for the next deposition cycle. For brevity, the following abbreviations will be used henceforth, ZnO IOs prepared after three ZnO deposition cycles and extracted twice with toluene: Z175-IO (ZnO IO prepared with PS175-T), Z70-IO (ZnO IO prepared with PS70-T), Z60-IO (ZnO IO prepared with PS60-T), and Z48-IO (ZnO IO prepared with PS48-T).

### 2.6. Assembly of ZnO Inverse Opal Thin Films

After drying, the PS template films assembled according to Section 2.4 were mineralized with 3 ZnO deposition cycles. The PS particles were removed from the PS/ZnO hybrid structure either through calcination at 500 °C for 3 h (heating rate: 1 K/min) or through extraction with toluene. For extraction, the substrate was stirred in toluene at 60 °C for 1 h and thoroughly washed with copious amounts of fresh toluene. This procedure was repeated once and the sample was dried under N_2_. Cross-section SEM images of all the synthesized IOs were used to calculate the mean pore size. The pore size distribution and standard deviations were calculated by using a Gaussian fit. The synthesis of Z175-IO was repeated three times. 243 pores were analyzed in total from these three experiments, and the average pore size, polydispersity and standard deviation were calculated as described above. The assembly of Z70-IO was repeated five times, and 285 pores were used for the pore size calculations. The synthesis of Z60-IO was repeated five times, and 283 pores were analyzed. For further reduction of the pore size, the as-obtained inverse replica (Z70-IO) was subjected to one or two additional mineralization cycles.

### 2.7. Assembly of Multilayered ZnO Inverse Opals

Multilayered ZnO IOs were prepared on plasma cleaned round microscope glass slides (Paul Marienfeld GmbH, Lauda-Königshofen, Germany) with a diameter of 25 mm and a thickness of 170 µm. One side of the slides was covered with a protecting foil during the convective assembly process. The first PS/ZnO hybrid layer was assembled as follows: A 25 µL droplet of ~10 wt% PS (*d* ~70 nm) was drop-cast on the exposed face of the glass slide. The droplet was then withdrawn at a speed of 0.3 mm/min. The assembly was performed at a temperature of 23°C and a relative environmental humidity of 37%. After assembly, the glass slides were dried under N_2_ and subjected to two 2 ZnO deposition cycles. The entire protocol was repeated twice where instead of 25 µL, 60 µL of the PS colloidal dispersion was taken for the convective assembly of the PS particles onto the previously deposited PS/ZnO layer. The PS particles were then removed using extraction with toluene as described in Section 2.6.

### 2.8. Characterization

The morphology of the ZnO IOs samples was characterized using a Zeiss Merlin Scanning Electron Microscope (SEM) operating at 3 kV and a working distance of 5 mm. Prior to analysis, the samples were sputtered with 5 nm of iridium. The PS particles, sputtered with 10 nm iridium, were characterized on a Gemini SEM 500 from Carl Zeiss Microscopy (Jena, Germany) at 3 kV and working distance of 7 mm. Using the software ImageJ, at least 150 particles or pores were evaluated (more details in Section 2.6). By describing the size distribution with a Gaussian function, the mean diameter *d* and the standard deviation σ were obtained. The polydispersity was calculated according to p = σ/d. UV-Vis measurements were performed on a Cary 5000 UV-Vis-NIR spectrometer (Varian, Darmstadt, Germany). Angle-resolved UV-Vis spectra were recorded with a universal measurement accessory (UMA). X-Ray diffractograms were obtained using a Rigaku Smartlab system (Neu-Isenburg, Germany) equipped with a Cu-Kα radiation source (λ = 1.54 Å). All optical measurements were performed at room temperature. Confocal Laser Scanning Microscopy (CLSM) and Fluorescence Correlation Spectroscopy (FCS) experiments were performed on a commercial device, LSM 880 (Carl Zeiss, Jena, Germany). The excitation was done with the 488 nm line of an Argon laser focused into the studied samples through a C-Apochromat 40×/1.2 W water immersion objective (Carl Zeiss, Jena, Germany). The emission was collected with the same objective and after passing through a confocal pinhole (set to 1 Airy unit), directed to a spectral detection unit (Quasar, Carl Zeiss, Jena, Germany) in which a detection range 500–550 nm was selected. A glass coverslip with the deposited ZnO IO layer was mounted in an Attofluor cell chamber (Thermo Fisher Scientific, Dreieich, Germany) and 600 µL of ~1 nM aqueous solution of Alexa Fluor 488 was added. As the inverse opal layer was relatively thin and had some cracks and defects, a CLSM was performed first in order to identify appropriate position for the FCS measurement. At each such position a series of 10 FCS measurements with a total duration 5 min were performed. The time-dependent fluctuations of the fluorescent intensity *δI(τ)* were recorded and analyzed by an autocorrelation function *G(τ)* = 1 *+ δI(τ)* · *δI(τ + τ)* *>/<I(τ)>*^2^. For an ensemble of *m* different types of freely diffusing fluorescence species, the autocorrelation function takes the following analytical form:

(1) $$G\left(\tau \right)=1+\left[1+\frac{f_{T}}{1-f_{T}}e^{-\tau /\tau_{T}}\right]\frac{1}{N}\overset{m}{\underset{i=1}{\sum }}\frac{f_{i}}{\left[1+\frac{\tau }{\tau_{Di}}\right]\sqrt{1+\frac{\tau }{S^{2}\tau_{Di}}}}$$

Here, *N* is the average number of diffusing fluorescence species in the observation volume, *f_T_* and *τ_T_* are the fraction and the decay time of the triplet state, *τ_Di_* is the diffusion time of the *i-th* type of species, *f_i_* is the fraction of component *i*, and *S* is the so-called structure parameter, *S* = *z*_0_/*r*_0_, where *z*_0_ and *r*_0_ represent the axial and radial dimensions of the confocal volume, respectively. Furthermore, the diffusion time, *τ_Di_*, is related to the respective diffusion coefficient, *D_i_*, through: $\tau_{Di}=\frac{r_{0}^{2}}{4D_{i}}$. The experimentally obtained *G*(*τ*) were fitted with Equation (1) yielding the corresponding diffusion times, and subsequently the diffusion coefficients of the fluorescent species. As the value of *r*_0_ depends strongly on the specific characteristics of the optical setup, a calibration experiments were performed using a fluorescent tracer with known diffusion coefficient, i.e., Alexa Fluor 488 in water.

## 3. Results and Discussion

### 3.1. Synthesis of ZnO IOs with Adjustable Pore Size

**Figure 1:**
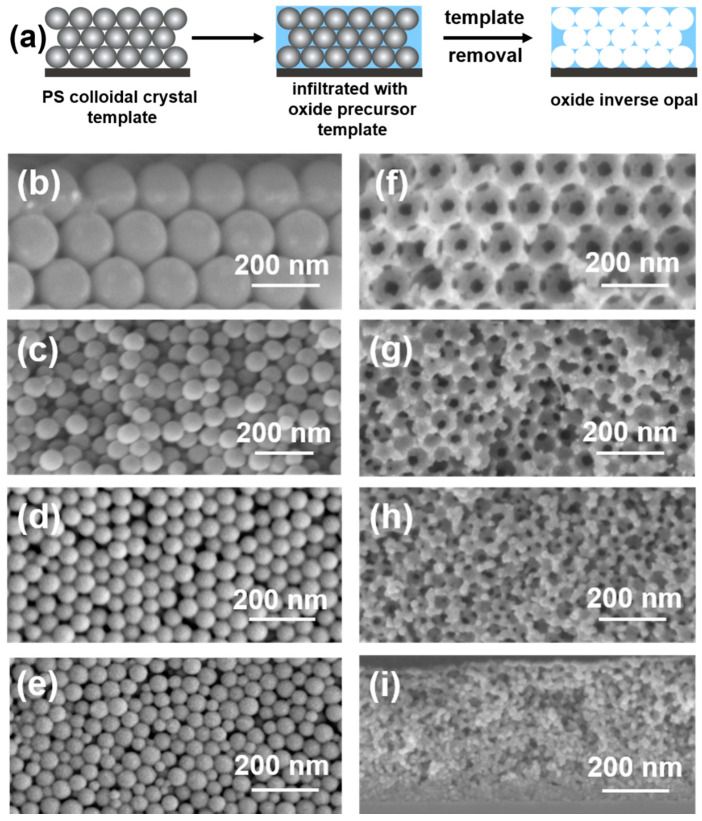
(**a**) Schematic representation of template-based ZnO inverse opal formation. (**b**–**e**) SEM images of polystyrene (PS) template films (**b**) PS175-T, (**c**) PS70-T, (**d**) PS60-T, and (**e**) PS48-T. (**f**–**i**). Cross-section SEM images of the corresponding porous ZnO IO films obtained after three ZnO deposition cycles and extraction with toluene at 60 °C. (**f**) Z175-IO, (**g**) Z70-IO, (**h**) Z60-IO, and (**i**) Z48-IO.

**Figure 2:**
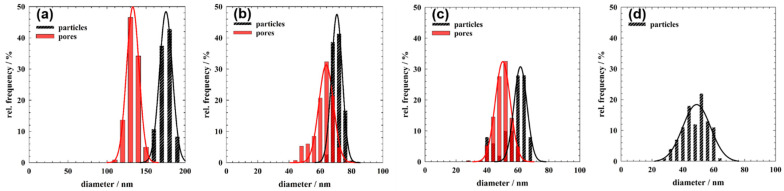
PS particle size distribution (black curve) and pore size distribution (red curve) in (**a**) PS175-T and Z175-IO, (**b**) PS70-T and Z70-IO, (**c**) PS60-T and Z60-IO and (**d**) PS48-T, respectively.

The synthesis of ZnO IOs is shown schematically in Figure 1a. In the first step, PS colloidal crystal template films (left) were infiltrated with a ZnO precursor solution, which were subsequently mineralized via CBD (centre). The sacrificial PS particles were then removed either by calcination or extraction with toluene (right). PS template films were prepared using PS particles with different diameters (175, 70, 60 and 48 nm), which were synthesized via emulsion polymerization [55]. The PS particles were arranged in a colloidal crystal film using convective assembly. By varying the withdrawal speed, the thickness of the PS films was adjusted to around 1 µm, which was sufficient and optimal to obtain stable template films for the subsequent mineralization (Supplementary Materials Figure S1). The PS template films were subjected to different number of deposition cycles (1–5 cycles) and extracted with toluene to obtain ZnO IO films. The impact of the number deposition cycles applied (amount of deposited material in the voids) on the stability and morphology of the IO films was initially examined. IOs obtained after one and two deposition cycles showed low integrity. These films did not maintain their shape and collapsed. However, the IOs obtained after three deposition cycles retained their spherical pore morphology irrespective of the size of PS particles in the template. Since the mineralization with four and five deposition cycles led to the formation of a ZnO film on top of the PS/ZnO hybrid film, all further experiments were conducted with three deposition cycles. For the XRD and UV-Vis measurements, the IOs obtained after three mineralization cycles were too thin to produce tangible results, and hence five deposition cycles were used. The SEM images in Figure 1b–e show the colloidal crystal template films of PS particles with different diameters, and their corresponding ZnO IOs, obtained after extraction with toluene are shown in Figure 1f–i. Figure 2 represents the distribution of particle and pore diameters for the template PS particles and their respective inverse replicas. This distribution was obtained from the analysis of cross-section SEM images using the software ImageJ by using a Gaussian distribution function (for more details, see Section 2.6 and Section 2.8). The corresponding values, i.e., the average diameters $d_{part}$ and $d_{pore}$, the standard deviations σ*_part_* and σ*_pore_*, the associated polydispersities $p_{part}$ and $p_{pore}$ as well as the relative pore size shrinkage (% shrinkage) are summarized in Table 2. As noted in Section 2.1 and Section 2.6, all the PS particle dispersions were synthesized once and the same solutions were used for the fabrication of all ZnO IOs, while the assembly of the corresponding ZnO IOs was repeated for several times. All the fabricated IOs showed volume shrinkage, i.e., the pore sizes of the ZnO IOs were smaller than the diameters of their corresponding PS template particles. This pore size shrinkage was more pronounced for the IO exhibiting the largest pore size. From XRD measurements discussed below, it can be seen that the ZnO phase mineralized in the voids of the opal template was crystalline. No post-synthetic treatment was therefore required to obtain crystalline ZnO. Hence, volume shrinkage due to morphological changes in the inorganic part appeared to be improbable. It is therefore posited that the reduction in the pore sizes primarily occurred during the drying process, which in large part was also responsible for the formation of cracks in the macrostructure.

The influence of the PS-particle diameter and polydispersity on the obtained ZnO IO films is discussed in the following paragraph in consonance with Figure 1. Large and monodisperse PS particles (175 nm) assembled in well-aligned arrays in the template film (Figure 1b). The corresponding ZnO IO film consisted of an ordered structure with regular interconnecting 131 nm sized pores (Figure 1f) with a similar polydispersity as the template PS particles. In order to ascertain the limits of this template-based approach towards fabrication of mesoporous IOs, smaller PS particles ($d_{part}$ < 100 nm) were used as templates. However, while the 175 nm particles exhibited a small polydispersity, the polydispersity increased strongly when the particle diameter was further reduced to 70, 60 and 48 nm. This increase in polydispersity can be attributed to an additional micellar nucleation mechanism, which appeared during the emulsion polymerization when the surfactant concentration approached the critical micelle concentration (CMC) [58]. As a consequence of the increased polydispersity, the long-range packing order in PS colloidal crystal template films was strongly reduced, as can be seen in the cross-section SEM images in Figure 1c–e. At the same time, the packing efficiency was enhanced [55]. However, even with short-range order, PS template films could yield ZnO IOs with open porosity. The spherical pore morphology in Z70-IO and Z60-IO (Figure 1e,g) was maintained and the pore size decreased. Thus, the upper boundary of the mesoporous range (2–50 nm) was reached with the synthesis of the Z60-IO with a pore size of 50 nm. The pore size distribution of the IOs became wider due to the increasing width of the PS particle size distribution. When the smallest PS particles (PS48-T template, Figure 1e) with the largest polydispersity of $p_{part}$ = 0.16 were used, the quality of the IO film was lost. The IO film thickness was lower compared to the template film thickness, and the spherical morphology of the pores could not be sufficiently retained. Although IO films with reduced pore sizes could be fabricated from smaller templates ($d_{part}$ < 50 nm), the issue of poor morphological integrity and polydispersity could not be mitigated. The structural disorder in mesoporous IOs can be attributed to the increased polydispersity of the template. As can be seen in Figure 1b,e, the size distribution of PS48-T was significantly higher than that of PS175-T. The smaller, irregular PS particles in PS48-T could intercalate in the voids of the larger particles and hinder the infiltration of the precursor solution. This in turn could result in lower infiltration depths in the template structure and reduced film thicknesses of the corresponding inverse replica. Additionally, the mismatch in the size of the PS particles strongly hampered the interconnectivity in the template structure, which had a direct impact on the quality of the ZnO inverse replica. From these observations, it can be concluded that the factor that limits the pore size and quality of the IO films is the polydispersity of the template. Although this template-based approach can be used to synthesize IO films with pore sizes in the mesoporous range, the existence of monodisperse template colloidal crystals is essential for achieving an ordered interconnected IO film with uniform pores with well-defined shapes.

### 3.2. Determination of the Infiltration Depth

**Figure 3:**
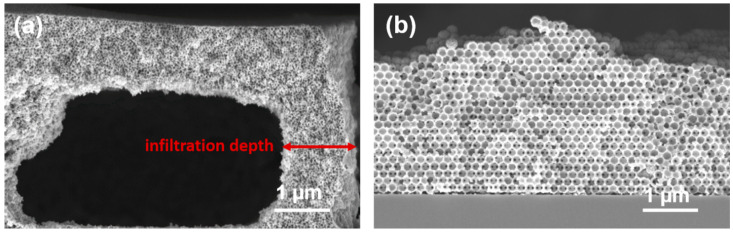
Cross-section SEM images of ZnO inverse opals obtained with (**a**) 60 nm sized PS particles and (**b**) 175 nm sized PS particles. Assembly conditions for both PS template films: 10 µL, 10 wt.% PS colloidal dispersion, withdrawal speed 0.3 mm/min, temperature 22.6 °C and humidity 53.8%, 3 ZnO mineralization cycles and solvent extraction.

An important issue which needs to be addressed during the mineralization of opaline templates is restricted infiltration. The use of thicker PS templates often results in incomplete or partial mineralization due to reduced infiltration of the ZnO precursor solution. Generally, the infiltration is restricted to the periphery of the film, whereas the bulk of the template remains largely undisturbed. Upon calcination or extraction, the PS particles that are not infiltrated by the precursor solution are removed, resulting in the formation of large fractures or cavities as shown in Figure 3a. A similar result was observed when PS-48T was mineralized with ZnO and was attributed to the increased polydispersity of the PS particles in the PS-48T template. In order to define the infiltration limits of sub-macroporous PS colloidal crystals, a systematic study was performed. Thus, PS-48T, PS-60T, PS-70T and PS-175T templates were summarily mineralized with 3 ZnO deposition cycles, and the infiltration depth was measured. For PS48-T, the infiltration depth was determined to be in the range of 230–700 nm. For PS-60T, infiltration depths were measured in the range of 1.1–1.4 µm. Similar results were obtained for PS-70T PS films, where the values were between 0.6 and 1.7 µm. For the PS templates discussed above, a film thickness of around 3 µm was sufficient to determine the infiltration depth. However, the cross-section SEM image of Zn175-IO (Figure 3b) synthesized from PS175-T shows complete ZnO infiltration of the template. To reconcile our earlier results with this new observation, thicker PS-175T template films had to be prepared. This was a difficult proposition since crack formation led to poor adhesion in micrometer-thick films (Figure S2). Additionally, the multiple washing steps warranted by the mineralization protocol resulted in the removal of parts of the template film from the substrate. This made it very difficult to convincingly assess the infiltration depth of PS-175T template films. Although the exact infiltration depth of PS175-T could not be measured, a justifiable trend is established for infiltration depths. With decreasing particle size, the polydispersity of the template becomes a major determinant of the infiltration depth. Templates with a large particle size distribution (such as PS48-T) suffer from restricted infiltration of the oxide precursor. Another important factor to be considered is the void volume of the template film. Infiltration is significantly inhibited when the void volume is reduced. As seen in PS175-T, larger void volumes result in unimpeded infiltration. A downward trend is observed with templates with smaller void volumes.

### 3.3. ZnO IOs with Different Pore Geometries

Calcination is a commonly used method to remove sacrificial polymeric template particles after mineralization. However, calcination can often result in drastic changes in the morphology and crystallinity of the inorganic phase in the IO. In order to examine the effect of calcination on the morphology of ZnO IOs, the template particles discussed above (PS48-T, PS60-T, PS70-T and PS175-T) were assembled into template thin films, mineralized with ZnO and then removed via calcination. The resultant IOs were compared with their extracted counterparts (Z48-IO, Zn60-IO, Z70-IO and Z175-IO). For accurate comparison, the number of deposition cycles used for calcination and extraction were kept the same (three deposition cycles).

**Figure 4:**
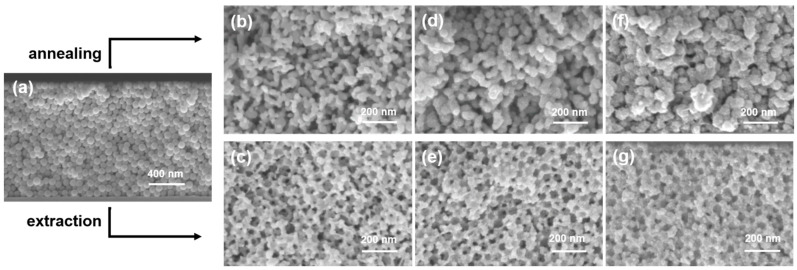
Cross-section SEM images of (**a**) PS particles with diameter 70 nm assembled in colloidal crystal thin film, (**b**) monomodal ZnO IO, obtained by mineralization of (**a**) with three ZnO deposition cycles and calcinated at 500 °C for 3 h, (**c**) monomodal ZnO IO obtained by mineralization of (**a**) with three ZnO deposition cycles and extraction of PS particles with toluene at 60 °C, (**d**) and (**e**) ZnO IOs from (**b**) and (**c**) further mineralized with one ZnO deposition cycle, respectively. (**f**) and (**g**) ZnO IOs from (**b**) and (**c**) further mineralized with two ZnO deposition cycles, respectively.

With PS175-T, both calcination and extraction yielded IOs which retained the morphology of their templates (Figure S3). However, after calcination a collapse of the IOs structure was observed (Figure S3a). This was attributed to the interplay of two factors: the sintering effect induced by the thermal treatment and the absence of a sufficient amount of ZnO, deposited with three deposition cycles, in the relatively big voids of the template. Calcination had a stronger impact on the morphologies of the IOs obtained from PS70-T (Figure 4). Upon extraction, the IOs obtained from PS70-T retained their parent spherical shape. However, upon calcination, gyroidal pores were observed in the resultant IO. A similar trend was observed with the IOs obtained from PS60-T (Figure S4). Hence, the sintering effect has a detrimental influence on the morphology of IOs obtained from smaller template particles (*d_part_* < 100 nm). Extraction with toluene is therefore required to preserve the morphological integrity of the template when operating in the mesoporous domain.

In an attempt to further reduce the pore size of the IOs and verify their open porosity, ZnO IOs prepared with 70 nm PS particles and with different pore geometries (spherical and gyroidal pores) were subjected to one and two additional ZnO deposition cycles. Roughening of the pore walls is visible in the cross-section images of both samples with different pore geometry (Figure 4d,e) implying the deposition of an additional ZnO layer on the pore wall. This effect is even more pronounced after two additional ZnO deposition cycles (Figure 4f,g). The cross-section SEM images have shown that the successive ZnO deposition on pore walls of the ZnO porous structures is possible. In addition, a qualitative reduction in the pore size of the IOs can also be seen. A quantitative determination of pore-size reduction requires additional specialized analytical techniques which are beyond the scope of our current investigation.

### 3.4. ZnO IOs Characterization

**Figure 5:**
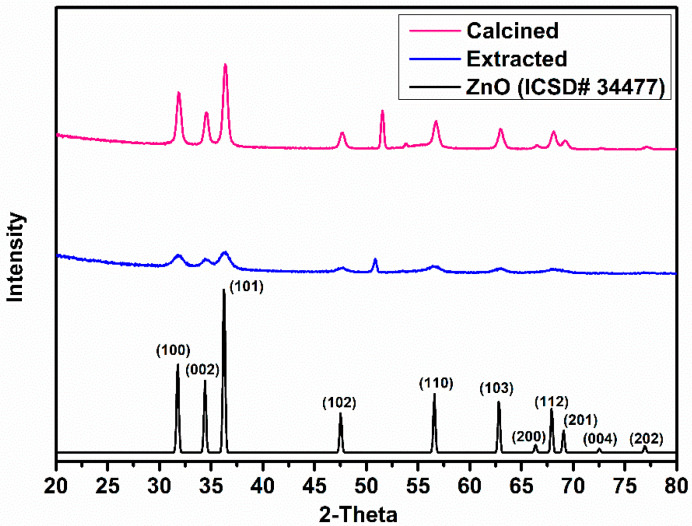
XRD diffractograms of ZnO IOs obtained from PS175-T template after five mineralization cycles. The technique used for template removal is indicated in the legend.

XRD diffractograms of ZnO IO films obtained from PS-175T after five mineralization cycles are shown in Figure 5. The observed peaks can be indexed to hexagonal wurtzite ZnO (ICSD deposition number: 34477) [59]. A strong correlation is observed for the (100), (002), (101), (102), (110), (103), (200), (112), (201), (004) and (202) planes. Evidently, the crystallinity of the IO films obtained by calcination is much higher than that of the IO films obtained by extraction. This increased crystallinity can be attributed to the formation of larger crystallites at the calcination temperature due to grain coalescence [60,61]. The peak at 52° could not be precisely indexed.

**Figure 6:**
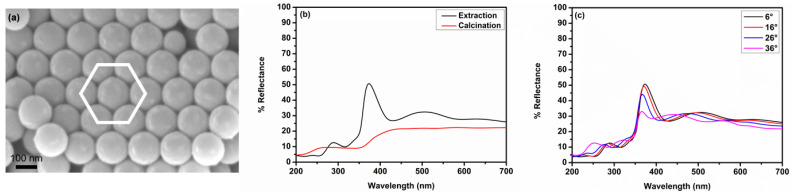
(**a**) SEM micrograph of PS-175T assembled using convective assembly showing the face-centered cubic arrangement of the particles. (**b**) Reflectance spectra of ZnO IOs obtained after five mineralization cycles using different methods for removal of the sacrificial PS particles and (**c**) Reflectance spectra of ZnO IOs (extracted) measured at different angles of incidence.

As detailed earlier, ZnO IOs are capable of producing photonic stop bands (PSBs). However, the refractive index of ZnO is not sufficiently large to produce a complete PSB [13]. As a result, ZnO photonic crystals often exhibit a partial PSB. The presence of a partial PSB impedes certain wavelengths of light from propagating through the crystal, thereby resulting in a periodic dielectric contrast. This is often manifested in the form of a sharp increase in reflectance due to enhanced Bragg diffraction. The existence of ordered pores in a close-packed arrangement is the key to achieve a PSB. Surface imperfections such as cracks and dislocations can lead to a deterioration in the optical properties of such photonic crystals. This in turn results in the absence of a discernible PSB. Determining the existence of a PSB can therefore provide preliminary information on the uniformity of the obtained IO film. The UV-Vis spectra of ZnO IO films obtained from PS175-T are shown in Figure 6.

For a 3-D photonic crystal, the position of the PSB can be approximated by using a modified form of the Bragg diffraction law [3].

(2) $$\lambda_{c}=2d\eta_{eff}$$

where, λ*_c_* is the position of the PSB, ‘*d*’ is the diameter of the template particles and $\eta_{eff}$ is the effective refractive index of the material. For a face-centered cubic (FCC) crystal, Equation (2) can be transformed into

(3) $$\lambda_{c}=2\sqrt{\frac{2}{3}}d\eta_{eff}$$

For, *d* = 175 nm, Equation (3) becomes,

(4) $$\lambda_{c}=286\eta_{eff}\;\mathrm{nm}\;$$

The effective refractive index of ZnO can be calculated using the following equation:

(5) $$\eta_{eff}^{2}=\eta_{ZnO}^{2}f+\eta_{air}^{2}\left(1-f\right)$$

where, $\eta_{ZnO}$ is the refractive index of ZnO thin films (1.6) [62] and f is the filling fraction of the FCC lattice. The theoretical value of f for an ideal FCC lattice is 0.74. For the inverted structure, the value of f becomes 1 − 0.74 = 0.26 [15]. By using these values, the value of $\eta_{eff}$ is determined to be 1.468. By substituting these values in Equation (4), the position of the PSB can be affixed at 420 nm. However, Equation (4) holds true only at normal incidence (i.e., the source beam is incident on the sample at 90°). Since the universal measurement accessory (UMA) uses an incidence angle of 6° to record the reflectance spectrum, Equation (4) must be transformed into an angle-dependent one. This can be achieved by using a modified form of the Bragg–Snell law [63].

(6) $$\lambda_{c}=2D\sqrt{\eta_{eff}-sin^{2}\theta }$$

where, $D=d\sqrt{\frac{2}{3}}$ is the interplanar spacing and θ is the angle of incidence. This expression enables us to calculate the positions of the PSB for different angles of incidence. From Equation (6), it also follows that the position of the PSB should be blue-shifted when the angle of incidence is increased. The results are tabulated in Table 3. It should be noted that these theoretical values are only applicable for an ideal IO with no volume shrinkage. The deviations in the positions of the angle-dependent stop bands can therefore be attributed to variations in the thickness of the film and volume shrinkage.

Figure 6a clearly shows the FCC arrangement of the PS colloidal crystal template. The PS particles were ordered in a close-packed structure with the (111) plane parallel to the substrate. As detailed earlier, the presence of an ordered periodic structure is essential for the existence of a PSB. The close-packed PS spheres were mineralized five times with the ZnO precursor and the template was removed by calcination or extraction to obtain the corresponding IO. From Figure 6b, the presence of a PSB at λ*_c_* = 373 nm was observed for the ZnO IOs obtained through extraction. Fabry–Perot (F-P) oscillations [64] were seen on each side of the stop band. No such stop band could be observed for the IOs obtained through calcination. This indicates that there was a significant structural disordering of the IO when the template was removed by calcination. This disordering was exacerbated by the sintering effect that often accompanies calcination [37]. These results were in good agreement with the SEM micrograph in Figure S3, which showed that the ordered spherical structure of the pores was lost upon calcination. From Figure 6c, the existence of angle-dependent stop bands for the ZnO IO obtained by extraction could be confirmed. With smaller templates such as PS-48T and PS-60T, the stop band lay beyond the UV-Vis-NIR observation range and hence could not be detected by the instruments used in this study.

### 3.5. Diffusion of Tracer Molecules in Multilayered ZnO IOs

The open porosity of the fabricated ZnO IOs enabled us to investigate the diffusion of small tracer molecules within the porous structure using fluorescence correlation spectroscopy (FCS). This method is based on evaluating the fluctuations of a fluorescence signal originating from fluorescent molecules diffusing in and out of a small (<1 µm^3^) probing volume created in the “focus” of a confocal microscope [65]. It is expected that pore reduction towards mesopore range, which is highly demanded for heterogeneous catalysis, would significantly influence the diffusion properties. To gain deeper insights into the diffusion properties of mesoporous IOs, ZnO IOs with spherical pores and a pore size of 60 nm were used. In order to achieve sufficiently thick films for analysis, ZnO IOs were assembled in multilayers as detailed in Section 2.6. 3 PS/ZnO hybrid layers were deposited on plasma-cleaned round microscope glass slides under optimized conditions. The thickness of each PS layer was tuned to around 1 µm to ensure complete infiltration of the ZnO precursor solution. Each layer was subjected to two ZnO deposition cycles to ensure that the top layer was not completely covered with ZnO. The presence of interconnecting pores at the interface is essential for the deposition of subsequent PS layers. At the end of the multilayer assembly, the PS particles were removed by extraction with toluene to obtain an IO with open porosity and spherical pores. The cross-section SEM images (Figure S5a) confirm the existence of a layered IO structure with well-preserved spherical pores.

**Figure 7:**
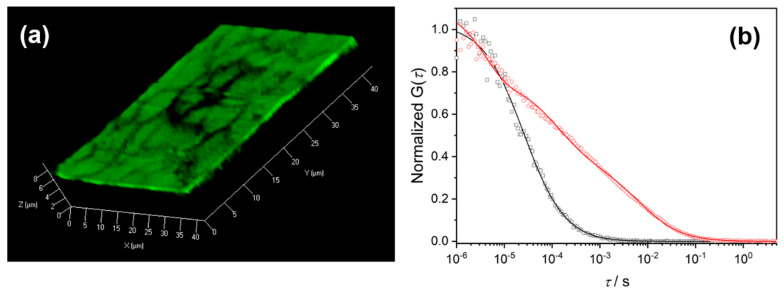
**a**) 3D confocal laser scanning microscopy (CLSM) image of multilayered ZnO inverse opal with a pore size of 60 nm, infiltrated with Alexa Fluor 488 aqueous solution. (**b**) Normalized autocorrelation functions obtained for Alexa Fluor 488 diffusing in bulk water (□) and in inverse opal structure (o). The solid lines represent the corresponding fits with Equation (1) (see experimental part) using *m* = 1 in the bulk water and *m* = 2 in the IO.

The diffusion of small fluorescent tracer molecules, i.e., Alexa Fluor 488 in the multilayered ZnO IO film and in bulk water (above the ZnO IO structure) was examined and compared. The tracer molecules were dissolved in an aqueous solution and allowed to infiltrate in the multilayered ZnO IO. The infiltration of the tracer molecules was confirmed via CLSM. The 3D CLSM image in Figure 7a shows the film-like structure of the multilayered ZnO IOs with domains that are separated from one other due to crack formation. CLSM imaging was further used to find an appropriate position with homogeneity in the IO structure at which the confocal probing volume was positioned for conducting the FCS measurements. Figure 7b represents the autocorrelation functions (ACFs) measured for Alexa Fluor 488 in the ZnO IO film (red curve) and in bulk water above the film (black curve). Compared to the ACF obtained for the diffusion of Alexa Fluor 488 in bulk water, the ACF acquired for the tracer molecules in the IO was shifted to longer decay-times. This attested to the slower diffusion of the tracer molecule within the pores. Furthermore, while the ACF measured in bulk water could be fitted well with a single diffusion component (*m* = 1 in Equation (1), experimental part, Section 2.7) with diffusion time *τ*_D,water_ = 25 µs, two diffusion components (*m* = 2 in Equation (1)) with diffusion times *τ*_D,1,ZnO_ = 125 µs and *τ*_D,2,ZnO_ = 7500 µs were needed to fit the ACF measured in the ZnO IO structure. The first diffusion process with *τ*_D,1,ZnO_ = 125 µs was roughly five times slower than the free diffusion in bulk water with *τ*_D,water_ = 25 µs. This diffusion process originated from tracers diffusing within the IO pores. As the lateral size of the FCS probing volume (~400 nm) was significantly larger than the pore diameter (~60 nm), the tracers had to diffuse through multiple interconnected pores in order to cross the observation volume and thus, their diffusion was not free, but restricted and slowed down by the pore walls. The observed ~5-fold diffusion slowdown corresponded to a diffusion coefficient of ~8.8 × 10^−11^ m^2^/s for the Alexa Fluor 488 molecules in the pores of the ZnO IO and was consistent with the results of earlier FCS studies that showed ~3-fold slowdown for the diffusion of the same tracer in silica IOs with spherical voids of 360 nm [46]. The increased diffusion slowdown in ZnO IOs (~5-fold) compared to that in silica IO (~3-fold) could be explained with the much smaller 60 nm sized pores in ZnO IO versus the 360 nm sized pores in silica IO and confirmed the significant impact of the pore size on the diffusion properties of molecules in porous materials. The second diffusion process observed in the ZnO IO had a diffusion time *τ*_D,2,ZnO_ = 7500 µs and thus was orders of magnitude slower than the diffusion in bulk water. In the case of silica IOs studied in [46], a similar slow diffusion process was observed and clearly attributed to the adsorption of the Alexa Fluor 488 molecules on the silica pore walls. Interestingly, the diffusion time measured here *τ*_D,2,ZnO_ = 7500 µs was significantly longer than *τ*_D,2,silica_ = 1200 µs measured in silica IOs. This can largely be attributed to the difference in surface chemical properties arising from a different oxide source. The isoelectric point (IEP) of silica was in the pH range 3.9 ± 0.5 [66], while the IEP of ZnO was at pH ~9.5 [67]. Considering the high negative charge of Alexa Fluor 488 [68], the attractive electrostatic interaction of the fluorescent tracer molecules with the positively charged ZnO surface in IO in the neutral aqueous solution should be much stronger than the interactions with the negatively charged pore walls of the silica IO.

## 4. Conclusions

In the first part of this work, ZnO IO films with tunable pore-sizes in the macro-mesoporous range were fabricated using the CBD method. PS particles with diameters of ~175, 70, 60 and 48 nm were assembled into opaline template films via convective assembly. The polymer templates were infiltrated with a ZnO precursor solution and mineralized. Solvent extraction of the template particles yielded ZnO IOs with open porosity and spherical pores. The pore size was reduced towards the mesoporous region by systematically decreasing the diameter of the PS template particles. Thus, ZnO IOs with an average pore diameter of 50 nm were successfully assembled. The infiltration depth of the ZnO precursor in the PS template films was significantly lowered with reduction of the template particle size due to the higher polydispersity of the PS particles and lower void volumes. Calcination-induced sintering led to a loss in spherical porosity in smaller template particles (PS70-T), resulting in the formation of IOs with gyroidal pores.

In the second part, ZnO IOs with a pore size of 60 nm were assembled into multilayered films, and the diffusion of a fluorescent tracer molecule (Alexa Fluor 488) through this porous structure was evaluated using CLSM and FCS. Two distinct diffusion times (*τ*_1_ = 125 µs (fast) and *τ*_2_ = 7500 µs (slow)) were observed within the IO film and ascribed to diffusion of the tracer through the pores and adsorption on the pore walls, respectively. Comparison with SiO_2_ IOs studied earlier showed that by reducing the pore size from 360 nm (for SiO_2_ IOs) to 60 nm (for ZnO IOs), a two-fold increase in the fast diffusion time (*τ*_1_) could be observed affirming the slower diffusion of the probe through the quasi-mesoporous ZnO IOs. The steep enhancement in the slow diffusion time (*τ*_2_) in ZnO IOs compared to SiO_2_ IO was attributed to the difference in the surface charge properties (such as surface polarity) of these oxides, which in turn results in a stronger interaction of the tracer molecule with the ZnO pore walls.

The presented data conclusively show that the pore size of ZnO IOs can be tuned towards the mesoporous domain, thereby offering an opportunity to design and fabricate mesoporous oxide IOs for tailored applications such as size-selective heterogeneous catalysis and photonics. In addition, CLSM and FCS have proven to be powerful techniques which can be used to determine the diffusion in confined structures and have great potential to enhance our understanding of molecular transport during catalytic processes.

## Figures and Tables

**Table 1:** A comparison of pore diameters of ZnO IOs fabricated using different techniques.

S.No	Template	Template Diameter (nm)	Method of Synthesis	IO Pore Diameter (nm)	Reference
1.	Polystyrene (PS)	270, 300 and 350	Sol-gel	~250–260	[16]
2.	PS	300	Oil-water interfacial assembly	280	[5]
3.	PS	230	Sol-gel	180	[11]
4.	Sulphonated PS	~80–120	Ultrasonic spray pyrolysis	~80	[14]
5.	PS	226	Chemical Bath Deposition (CBD)	148 ± 14	[55]

**Table 2:** Average diameters d, standard deviations σ and polydispersities *p* = σ/*d* of the template particles and their corresponding ZnO IO pores obtained after three mineralization cycles and extraction with toluene. The % shrinkage is also represented.

	Template			Inverse Opal		% Shrinkage
Particle Diameter (*d_part_*) (nm)	σ*_part_*(nm)	*p_part_*	Pore Diameter (*d_pore_*) (nm)	σ*_pore_* (nm)	*p_pore_*	
175	±9	0.05	131	±7	0.05	25
70	±4	0.05	60	±4	0.06	14
60	±4	0.07	50	±5	0.09	17
48	±8	0.16	-	-	-	-

**Table 3:** Variation in the position of the PSB with angle of incidence.

Angle of Incidence (θ)	Calculated PSB (nm)	Observed PSB (nm)
6	345	373
16	337	371
26	323	367
36	303	365

## Data Availability

The data presented in this study are available on request from the corresponding author.

## Supplementary Materials

The following are available online at https://www.mdpi.com/2079-4991/11/1/196/s1, Figure S1: Thickness of PS template films (PS particles with diameters 60 nm, 70 nm and 175 nm, respectively) obtained using convective assembly technique as a function of the withdrawal speed, Figure S2: Cross-section SEM image of a PS template film obtained with 175 nm sized PS particles, Figure S3: Cross-section SEM image of ZnO inverse opal obtained with 175 nm sized PS particles after 3 mineralization cycles applying (a) calcination and (b) solvent extraction of the PS template particles, Figure S4: Cross-section SEM image of ZnO inverse opal obtained with 60 nm sized PS particles after 3 mineralization cycles applying (a) calcination and (b) solvent extraction of the PS template particles, Figure S5: (a) Cross-section SEM image of multilayered ZnO IO used for confocal laser scanning microscopy (CLSM) and fluorescence correlation spectroscopy (FCS) analysis. (b) Fluorescence intensity scan through the ZnO IO film immersed in Alexa Fluor 488 water solution in direction normal to the film plane.
