# Supplementary material for: From Macro to Mesoporous ZnO Inverse Opals: Synthesis, Characterization and Tracer Diffusion Properties
Source: Nanomaterials (Basel). 2021 Jan 14;11(1):196. doi: 10.3390/nano11010196 (PMC7828802; doi:10.3390/nano11010196)
Supplement: Supplementary file 1 [file nanomaterials-11-00196-s001.pdf]

nanomaterials

MDPI

Supplementary Information

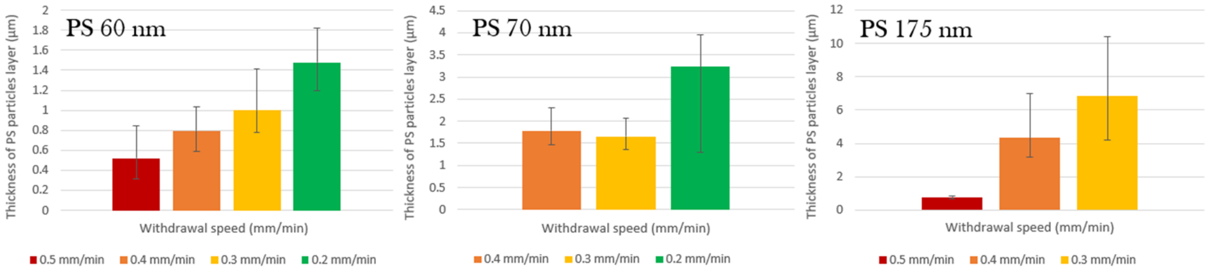

Figure S1. Thickness of PS template films (PS particles with diameters 60 nm, 70 nm and 175 nm, respectively) obtained using convective assembly technique as a function of the withdrawal speed.

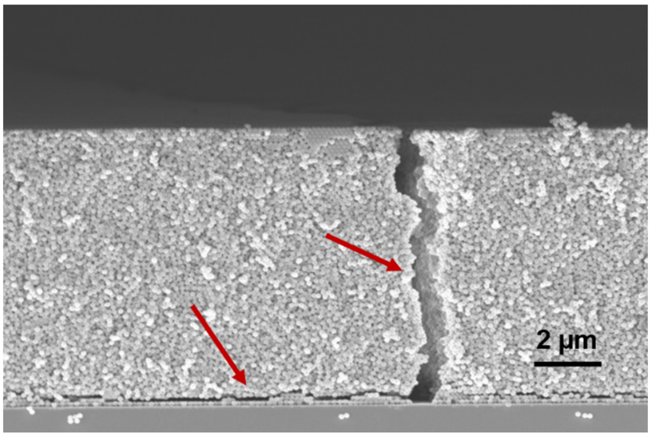

Figure S2. Cross-section SEM image of a PS template film obtained with 175 nm sized PS particles (assembly conditions: withdrawal speed 0.3 mm/min, temperature 24.5 °C and humidity 39.5 %). The crack formation during the drying process and the bad adhesion to the substrate result in removal of the film from the substrate by the multiple washing steps, required by the synthesis of the ZnO IOs, and make difficult the determination of the infiltration depth with the big PS particles.

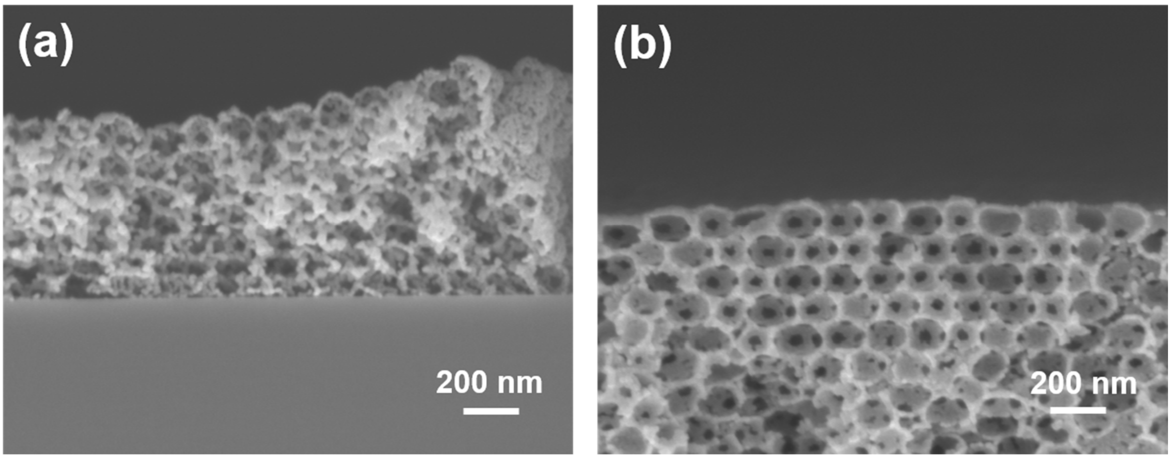

Figure S3. Cross-section SEM image of ZnO inverse opal obtained with 175 nm sized PS particles after 3 mineralization cycles applying (**a**) calcination and (**b**) solvent extraction of the PS template particles. Convective

assembly conditions for PS particles arrangement in the template film: 10 µl, 10% PS colloidal dispersion (d = 175 nm) withdrawal speed 0.3 mm/min, temperature 24.5 °C and humidity 39.5%.

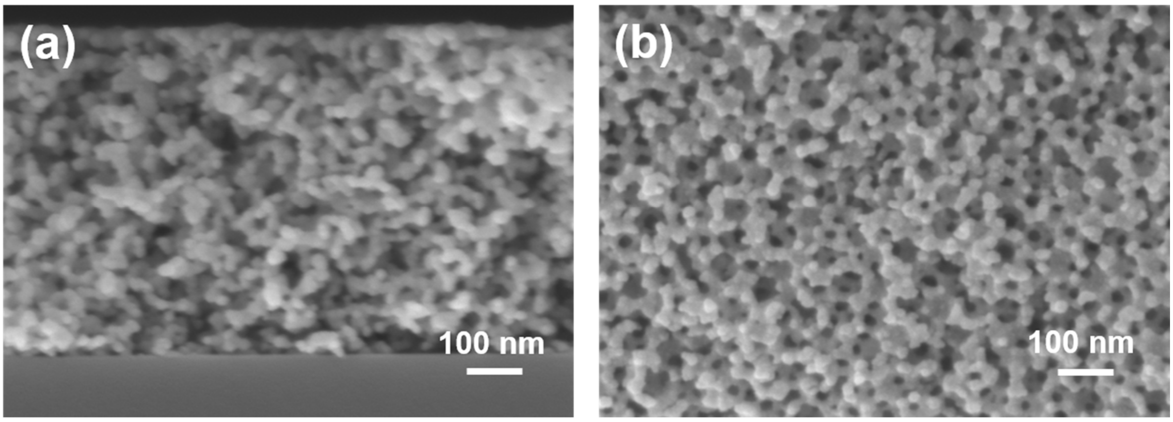

Figure S4. Cross-section SEM image of ZnO inverse opal obtained with 60 nm sized PS particles after 3 mineralization cycles applying a) calcination and b) solvent extraction of the PS template particles. Convective assembly conditions: 10 µl, 10 wt.% PS colloidal dispersion (d = 60 nm) (**a**) withdrawal speed 0.6 mm/min, temperature 22.2°C and humidity 50.3 %. (**b**) withdrawal speed 0.2 mm/min, temperature 22.5°C and humidity 54.6 %. The calcination of the PS/ZnO hybrid film resulted in porous film with gyroidal pores, while the by extraction with organic solvent, the spherical shape of the template particles is preserved in the inverse replica.

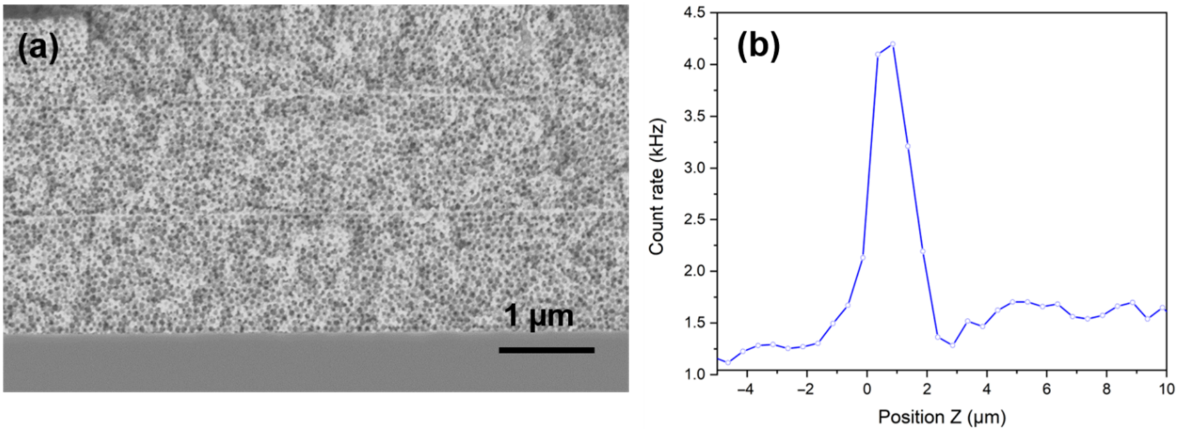

Figure S5. (**a**) Cross-section SEM image of multilayered ZnO IO used for confocal laser scanning microscopy (CLSM) and fluorescence correlation spectroscopy (FCS) analysis. (**b**) Fluorescence intensity scan through the ZnO IO film immersed in Alexa Fluor 488 water solution in direction normal to the film plane. z denotes the distance from the glass substrate.
